# The cost-effectiveness of digital health interventions: A systematic review of the literature

**DOI:** 10.3389/fpubh.2022.787135

**Published:** 2022-08-11

**Authors:** Andrea Gentili, Giovanna Failla, Andriy Melnyk, Valeria Puleo, Gian Luca Di Tanna, Walter Ricciardi, Fidelia Cascini

**Affiliations:** ^1^Section of Hygiene and Public Health, Department of Life Sciences and Public Health, Università Cattolica del Sacro Cuore, Roma, Italy; ^2^Department of Public Health, University of Verona, Verona, Italy; ^3^Statistics Division, The George Institute for Global Health, University of New South Wales, Newtown, NSW, Australia

**Keywords:** digital health, telemedicine, mobile health, electronic health, telehealth, digital care, cost-effectiveness

## Abstract

**Background:**

Digital health interventions have significant potential to improve safety, efficacy, and quality of care, reducing waste in healthcare costs. Despite these premises, the evidence regarding cost and effectiveness of digital tools in health is scarce and limited.

**Objectives:**

The aim of this systematic review is to summarize the evidence on the cost-effectiveness of digital health interventions and to assess whether the studies meet the established quality criteria.

**Methods:**

We queried PubMed, Scopus and Web of Science databases for articles in English published from January 1, 2016 to December 31, 2020 that performed economic evaluations of digital health technologies. The methodological rigorousness of studies was assessed with the Consolidated Health Economic Evaluation Reporting Standards (CHEERS). The review was conducted according to the Preferred Reporting Items for Systematic Reviews and Meta-Analysis (PRISMA) 2009 checklist.

**Results:**

Search identified 1,476 results, 552 of which were selected for abstract and 35 were included in this review. The studies were heterogeneous by country (mostly conducted in upper and upper-middle income countries), type of eHealth intervention, method of implementation, and reporting perspectives. The qualitative analysis identified the economic and effectiveness evaluation of six different types of interventions: (1) seventeen studies on new video-monitoring service systems; (2) five studies on text messaging interventions; (3) five studies on web platforms and digital health portals; (4) two studies on telephone support; (5) three studies on new mobile phone-based systems and applications; and (6) three studies on digital technologies and innovations.

**Conclusion:**

Findings on cost-effectiveness of digital interventions showed a growing body of evidence and suggested a generally favorable effect in terms of costs and health outcomes. However, due to the heterogeneity across study methods, the comparison between interventions still remains difficult. Further research based on a standardized approach is needed in order to methodically analyze incremental cost-effectiveness ratios, costs, and health benefits.

## Introduction

In a rapidly evolving society, the progress of digital technology used to improve human health and well-being needs to be constantly evaluated, both in its effectiveness and its efficiency. The WHO defines eHealth as “the cost-effective and secure use of information and communications technologies in support of health and health-related fields, including health-care services, health surveillance, health literature, and health education, knowledge and research” (1).

Digital technology encompasses many areas of eHealth, such as e-learning, telemedicine, mobile health and health information systems. eHealth also benefits from progress in related fields, such as artificial intelligence, big data analytics and genomics.

Digitized health-related data is easier to store and quickly analyze, especially when structuring a data-driven approach to build analytical models for safety improvement, managing clinical risk and increasing the quality of healthcare organizations (2).

During the COVID-19 pandemic, digital health technologies were successfully implemented to aid contact tracing, isolation management, primary care improvement and communication between citizens and decision makers (3).

South Korea is a prime example of a country with widespread digital health implementation, where remotely located supercomputers are used to secure and analyze medical big data and about 50% of digitized hospitals already use a paperless and comprehensive health care system. A rapid response and a cutting-edge government-run digital contact tracing system allowed South Korea to have early success in flattening the curve during its first wave of COVID-19 (4).

Today more than 120 countries are prioritizing health-related digital progress, with a growing need to systematically implement standards-based interoperable solutions.

Despite the institutional fervor and wide applicability of digital health strategies, healthcare facilities and services are struggling to assess the cost-effectiveness of different solutions. The absence of standards and tools for the comparative assessment of functionality and value of fast-evolving digital health solutions exacerbates the pressing need for quality evidence to navigate normative change (5).

This systematic review aims to describing the cost effectiveness of digital health interventions by assessing their impact on standardized indicators, such as Quality Adjusted Life Years (QALYs) and Disease Adjusted Life Years (DALYs), on healthcare expenditure by comparing the strategies with the Median-Based Incremental Cost-Effectiveness Ratio (ICER), and by evaluating the quality of the evidence reported.

## Methods

### Search strategy

A systematic review of relevant articles published on the cost-effectiveness of digital health technologies was developed in March 2021. The researchers developed the search strategies from January to February 2021 to include a wide range of digital health innovations. The academic databases and systems inquired were PubMed, Web of Science and Scopus, using the query reported in Appendix 1. A manual search of reference lists of both relevant systematic reviews and included studies was also performed. Detailed information and query strings used for the search are disclosed the manuscript.

The systematic literature review was conducted according to the Preferred Reporting Items for Systematic Reviews and Meta-Analysis (PRISMA) 2009 checklist.

### Inclusion/exclusion criteria

Eligible studies included any original study (cohorts, clinical trials, cross-sectional studies, case-control studies and case series) that reported an analysis of the cost effectiveness of digital health applications and innovations, with or without a comparison to standard care. Due to the rapidly changing nature of digital technologies and because a similar systematic review was released in 2015 (6), only articles published from January 1, 2016 to December 31, 2020 and written in English were included. Unavailable full texts, abstract-only papers, case reports and secondary research (commentaries, editorials, etc.) were excluded from the study. An exclusion criterion included studies where the digital health innovation was only used for recording patients' information. More detailed information is contained in the PICO criteria table (Table 1).

**Table 1:** Population, Intervention, Comparison, Outcome (PICO) inclusion and exclusion criteria.

	**Include**	**Exclude**
Population	• Users using any form of digital health implementation	• Not applicable
Intervention & Comparator	• Not applicable	• Not applicable
Outcomes	• All reported outcomes related to cost-efficacy analysis of digital health technologies will be included	• Clinical outcomes
Study design	• Any original study (cohorts, clinical trials, cross-sectional studies, case-control studies, and case series) • Reviews (To be included in the discussion)	• Unavailable full texts • Abstract-only papers •Case reports•Secondary research (commentaries, editorials, etc.)
Limits	English language only Articles published in peer reviewed journals only	
Timespan	01/01/2016–31/12/2020	

### Selection process and data extraction

The results of the electronic search were downloaded into a reference manager library (EndNote). After duplicates were removed, titles and abstracts were reviewed by 2 experienced systematic reviewers working independently to determine whether each study met the eligibility criteria. Full-text copies of potentially relevant studies were retrieved and further assessed against inclusion/exclusion criteria by two independent reviewers. At both stages, disagreements were resolved by discussion or a third reviewer. At the end of the full-text review, the articles that met all predefined criteria were read by two researchers to confirm the inclusion of these articles.

A pilot data extraction was conducted by two of the investigators. Any discrepancy pertinent to data extraction was discussed to reach a consensus. The collected information included the following items: (1) general information (including authors, publication date, title, and country); (2) study characteristics (including discipline examined and kind of intervention); (3) methodology (including modeling method, time horizon, and perspective); (4) cost-effectiveness information (including cost measurement, consequence measurement, and ICER); and (5) key findings (and conclusion).

### Assessment of methodological quality

The Consolidated Health Economic Evaluation Reporting Standards (CHEERS) checklist, developed by the International Society for Pharmacoeconomics and Outcomes Research, was the questionnaire used to assess the methodological quality of each study included at the end of the selection process. The CHEERS checklist included 24 items, and the recommendations were subdivided into six categories: (1) title and abstract, (2) introduction, (3) methods, (4) results, (5) discussion, and (6) other. One point was assigned to each item when the quality criteria were fulfilled (and zero points for not entirely conforming to the criteria) to generate a total score (maximum score is 24).

Two of the investigators independently assessed the quality of each study and assigned the scores based on the CHEERS checklist. Any disagreement was resolved by discussion and consensus with a third investigator.

## Results

### Search results

**Figure 1:**
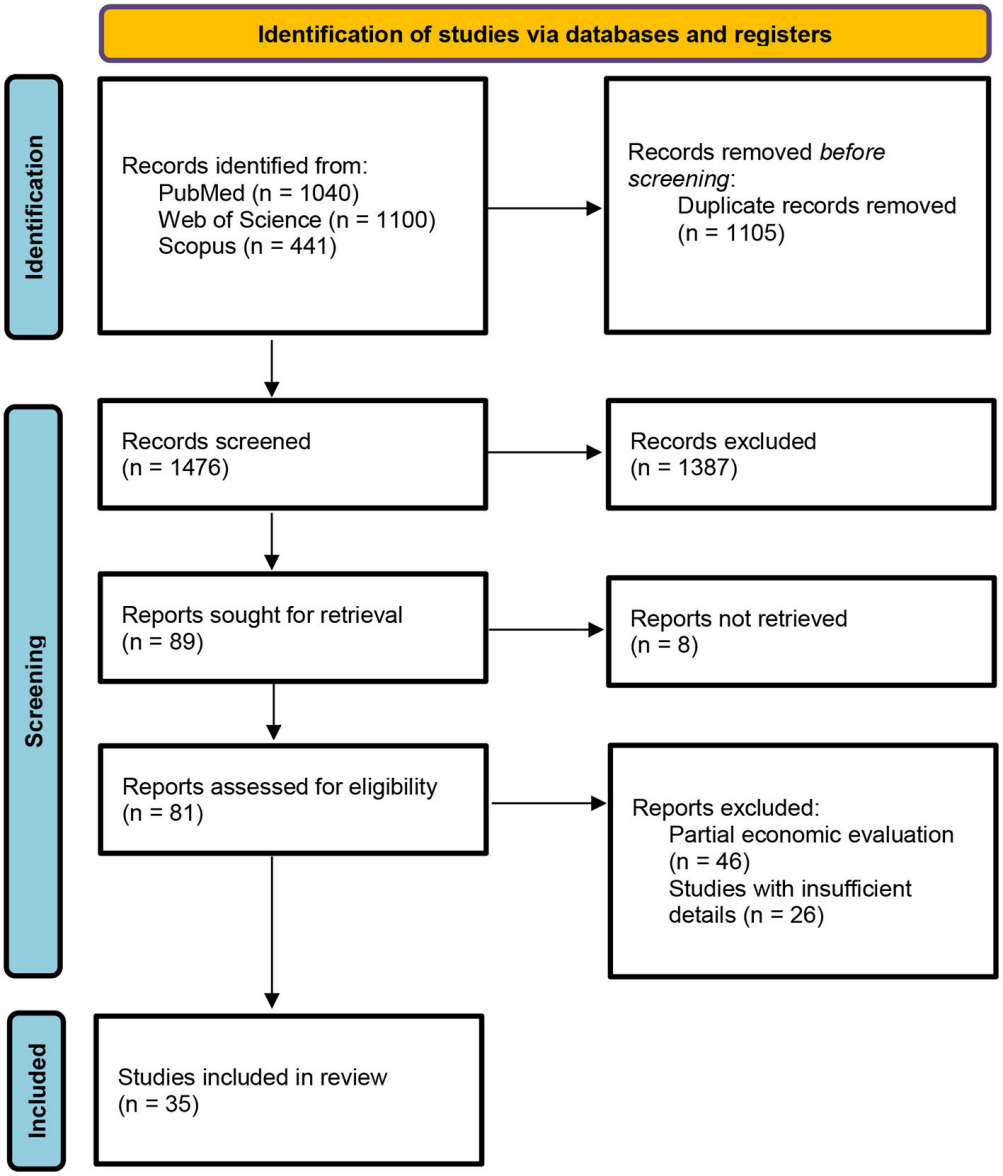
PRISMA statement flow diagram.

The results of the data extraction and selection process are shown in Figure 1. The database search, after duplicates were removed, returned a total of 1,476 records. In compliance with inclusion/exclusion criteria, the screening by title determined the inclusion of 552 abstracts. The abstract screening reported the inclusion of 81 full-text articles. Following the Preferred Reporting Items for Systematic Reviews and Meta-Analyses (PRISMA) guidelines, a total of 35 out of the 81 articles were included in the final review.

### Study characteristics

Records information and study characteristics have been subsequently extracted and summarized in Table 2.

**Table 2:** Study characteristics.

**Author (published year)**	**Country**	**Discipline**	**Study design**	**Perspective**	**Economic evaluation type**	**Quality assessment (CHEERS)**	**Sample size**
Clarke et al. (2018) (7)	England	Pneumology	RCT	Health service perspective	Cost-effectiveness analysis	18/24 (75%)	227
Krishnan et al. (2019) (8)	USA	Primary care	2-arm parallel-group randomized controlled trial	Healthcare payer perspective	Cost-effectiveness analysis	21/24 (88%)	194
Lugo et al. (2019) (9)	Spain	Pneumology	Prospective, open, randomized study	Healthcare payer perspective	Cost-effectiveness analysis	17/24 (71%)	186
Wan et al. (2019) (10)	USA	Endocrinology	Prospective pragmatic trial	Societal perspective	Cost-effectiveness analysis	19/24 (79%)	81
Islam et al. (2020) (11)	Bangladesh	Endocrinology	Prospective, randomized, controlled trial	Health service system perspective	Cost-effectiveness analysis	21/24 (88%)	236
Oostingh et al. (2019) (12)	Netherlands	Gynecology	RCT	Healthcare and societal perspective	Cost-effectiveness analysis	19/24 (79%)	793
Song et al. (2018) (13)	Japan	Gynecology	RCT	Health insurance society and companies' perspective	Cost-effectiveness analysis	22/24 (92%)	1,526
Lowry et al. (2020) (14)	USA	Gynecology	Comparative modeling study	Federal payer perspective	Cost-effectiveness analysis	19/24 (79%)	Not specified
Bahrainwala et al. (2020) (15)	Madagascar	Infectious diseases	Modeling study	Healthcare perspective	Cost-effectiveness analysis	21/24 (88%)	500
Thakar et al. (2018) (16)	India	Neurology	Prospective cohort	Societal perspective	Cost-effectiveness analysis	19/24 (79%)	1,200
Whetten et al. (2018) (17)	USA	Neurology	RCT	Healthcare payer perspective	Cost-effectiveness analysis	21/24 (88%)	777
Nguyen et al. (2016) (18)	Singapore	Ophtalmology	Prospective Cohort	Health system and societal perspectives	Cost-effectiveness analysis	23/24 (96%)	757
Buvik et al. (2019) (41)	Norway	Orthopedy	RCT	Societal and health sector perspective	Cost-effectiveness analysis	18/24 (75%)	389
Vestergaard et al. (2020) (19)	Denmark	Primary care	RCT	Danish public healthcare sector perspective	Cost-effectiveness analysis	21/24 (88%)	274
Oksman et al. (2017) (20)	Finland	Primary care	RCT	Not specified	Cost-effectiveness analysis	15/24 (63%)	1,535
Levy et al. (2017) (21)	USA	Primary care	RCT	Primary health system perspective	Cost-effectiveness analysis	21/24 (88%)	8,544
Kumar et al. (2018) (22)	USA	Primary care	Prospective cohort	Societal and payer perspective	Cost-effectiveness analysis	23/24 (96%)	100,000
Jo et al. (2019) (23)	Bangladesh	Primary care	RCT	Program perspective (inclusive of development, start-up, and implementation phases)	Cost-effectiveness analysis	21/24 (88%)	610
Cleghorn et al. (2019) (24)	New Zealand	Primary care	RCT	Health system perspective	Cost-effectiveness analysis	23/24 (96%)	4,400,000
Prinja et al. (2018) (25)	India	Primary care	RCT	Both health system and societal perspective	Cost-effectiveness analysis	22/24 (92%)	300,000
O'Sullivan et al. (2020) (26)	Ireland	Primary care	RCT	Publicly-funded healthcare system perspective	Cost-effectiveness analysis	22/24 (92%)	565
Nordyke et al. (2019) (27)	USA	Primary care	Prospective cohort	Healthcare payer perspective	Cost-effectiveness analysis	21/24 (88%)	5,145
Painter et al. (2017) (28)	USA	Psychiatry	RCT	Societal perspective	Cost-effectiveness analysis	21/24 (88%)	265
de Jong et al. (2020) (29)	Netherlands	Gastroenterology	RCT	Societal perspective	Cost-effectiveness and cost-utility analysis	21/24 (88%)	909
Hoyo et al. (2019) (30)	Spain	Gastroenterology	RCT	Societal perspective	Cost-effectiveness and cost-utility analysis	22/24 (92%)	63
Fusco and Francesco (2016) (31)	Italy	Orthopedy	Modeling study	Health sector and society	Cost-effectiveness and cost-utility analysis	22/24 (92%)	1,000
Watson et al. (2018) (32)	USA	Psychiatry	RCT	Third party-payor perspective and a partial societal perspective	Cost-effectiveness and cost-utility analysis	17/24 (71%)	179
Yoo et al. (2016) (33)	USA	Intensive care	Modeling study	Healthcare perspective	Cost-effectiveness	21/24 (88%)	Not specified
Witt Udsen et al. (2017) (34)	Denmark	Pneumology	Cluster-randomized trial	Healthcare and social care sector perspective (including hospital services, primary care, medicine, home care services and rehabilitation)	Cost-effectiveness and cost-utility analysis	22/24 (92%)	1,225
Romero-Sanchiz et al. (2017) (35)	Spain	Psychiatry	Pragmatic, multi-center, three- armed parallel RCT	Societal perspective	Cost-effectiveness and cost-utility analysis	24/24 (100%)	296
Wang et al. (2016) (36)	China	Primary care	RCT	Societal and health payer perspective	Cost-effectiveness analysis	17/24 (71%)	11,987
Lopez-Villegas et al. (2020) (37)	Norway	Cardiology	Open-label, 1:1, randomized, non-masked, controlled trial	National Healthcare System and Societal perspective	Cost-effectiveness and cost-utility analysis	21/24 (88%)	50
Sjostrom et al. (2017) (38)	Sweden	Gynecology	RCT	Societal perspective	Cost-effectiveness and cost-utility analysis	22/24 (92%)	123
Modi et al. (2020) (39)	India	Pediatrics	Two-arm, parallel, stratified cluster randomized controlled trial	Program perspective	Cost-effectiveness analysis	21/24 (88%)	561
Sharifi et al. (2017) (40)	USA	Primary care	RCT	Societal perspective	Cost-effectiveness analysis	22/24 (92%)	2,000,000

The 35 studies spanned 17 countries, the majority of which were conducted in high income economies (28, 80%), compared to 7 in low-income economies (1, 2.9%), lower-middle income economies (5, 14.3%) and upper-middle income economies (1, 2.9%).

The most represented country was the United States of America, with 11 studies (31.4%).

The articles covered a wide range of disciplines: 12 studies discussed primary care (34.3%); three discussed gynecology (11.4%); pneumology and psychiatry had three studies each (8.6%); endocrinology, gastroenterology, neurology and orthopedy had two studies each (5.7%); cardiology, infectious disease, ophthalmology and pediatrics had one study each (2.9%).

The types of digital health intervention implemented to support citizens and patients and to reduce costs from both societal and health payer perspective, were:

- Video-conferencing system: 17/35, 48.6% (7, 9, 10, 16–20, 28–31, 33, 34, 36, 37, 41).
- Text messaging intervention: 5/35, 14.3% (11, 12, 23, 24, 26).
- Web platforms and digital health portals: 5/35, 14.3% (27, 32, 35, 38, 40).
- Telephone support: 2/35, 5.7% (8, 21).
- Mobile phone-based systems and applications: 3/35, 8.6% (13, 22, 39).
- Digital technologies and innovations: 3/35, 8.6% (14, 15, 25).

Cost-effectiveness analysis (CEA) was a criterion of selection, therefore all the studies included a CEA, whereas a cost-utility analysis (CUA) has been performed in addition to the primary CEA in eight studies (22.9%) (29–32, 34, 35, 37, 38).

The costing perspective was mostly from payer/program/health service provider perspective (24, 68.7%), whereas the costing perspective was societal in 19 studies (54.3%). Only one study did not clearly report the costing perspective (2.9%).

In order to obtain QALYs, the studies of this review referred to different instruments deriving from the trials of reference. The questionnaires and surveys used to estimate QALYs were: ShortForm-6 Dimensions (32); a multistate body mass table model (24); European Quality of Life 5-Dimensions (9–11, 19, 29, 30, 33, 34, 37, 41); Health Related Quality of Life (8, 14, 20, 31); Generalized Anxiety Disorder 7 (22); Quebec Sleep Questionnaire and Epworth Sleepiness Scale (9); Quality of Well-being Scale and Short Form Health Survey for Veterans (28); International Consultation on Continence Modular Questionnaire on Lower Urinary Tract Symptoms and Quality of Life (38). For the rest of the articles, QALYs were estimated from existing literature.

### Study quality

The number of items of the CHEERS Checklist satisfied by each study and the relative percentage are shown in Table 3.

**Table 3:** Consolidated health economic evaluation reporting standards checklist.

**Section item**	**Clarke et al. (7)**	**Wang et al. (36)**	**Sharifi et al. (40)**	**Sjostrom et al. (38)**	**Song et al. (13)**	**Vestergaard et al. (19)**	**Thakar et al. (16)**	**Painter et al. (28)**	**Prinja et al. (25)**
Title	1	1	1	1	1	1	1	1	1
Abstract	1	0	1	1	1	1	1	1	1
Background and objectives	1	1	1	1	1	1	1	1	1
Target population and subgroups	1	0	1	1	1	0	0	0	0
Setting and location	1	1	1	1	1	1	1	0	1
Study perspective	1	1	1	1	1	1	1	1	1
Comparators	1	1	1	1	1	1	1	1	1
Time horizon	1	1	1	1	1	1	1	1	1
Discount rate	0	0	1	1	1	1	0	1	1
Choice of health outcomes	1	1	1	1	1	1	1	1	1
Measurement of effectiveness	0	0	1	1	1	1	1	1	0
Measurement and valuation of preference based outcomes	0	0	1	1	1	1	1	1	1
Estimating resources and costs	1	1	1	1	1	1	1	1	1
Currency, price date, and conversion	0	1	1	1	0	1	0	1	1
Choice of model	0	0	1	0	1	0	1	0	1
Assumptions	1	1	1	1	1	1	1	0	1
Analytical methods	1	1	1	1	1	1	1	1	1
Study parameters	1	1	1	1	1	1	1	1	1
Incremental costs and outcomes	1	1	1	1	1	1	1	1	1
Characterizing uncertainty	1	1	0	1	1	1	1	1	1
Characterizing heterogeneity	0	0	0	0	0	0	0	1	1
Study findings, limitations, generalizability, and current knowledge	1	1	1	1	1	1	1	1	1
Source of funding	1	1	1	1	1	1	0	1	1
Conflicts of interest	1	1	1	1	1	1	1	1	1
Total	18	17	22	22	22	21	19	20	22
Ratio	0.75	0.71	0.92	0.92	0.92	0.88	0.79	0.83	0.92
Quality	Fair	Fair	Good	Good	Good	Good	Fair	Good	Good
**Section item**	**Levy et al**. **(****21****)**	**Kumar et al**. **(****22****)**	**Nguyen et al**. **(****18****)**	**Nordyke et al**. **(****27****)**	**O'Sullivan et al**. **(****26****)**	**Jo et al**. **(****23****)**	**Whetten et al**. **(****17****)**	**Cleghorn et al**. **(****24****)**	**Islam et al**. **(****11****)**
Title	1	1	1	1	1	1	1	1	1
Abstract	0	1	1	1	1	0	1	1	0
Background and objectives	1	1	1	1	1	1	1	1	1
Target population and subgroups	1	1	1	0	1	0	0	0	0
Setting and location	1	1	1	0	1	1	1	1	1
Study perspective	1	1	1	1	1	1	1	1	1
Comparators	1	1	1	1	1	1	1	1	1
Time horizon	1	1	1	1	1	1	1	1	1
Discount rate	1	1	1	1	1	1	0	1	1
Choice of health outcomes	1	1	1	1	1	1	1	1	1
Measurement of effectiveness	1	1	1	1	1	1	0	1	1
Measurement and valuation of preference based outcomes	1	1	1	1	1	0	1	1	1
Estimating resources and costs	1	1	1	1	1	1	1	1	1
Currency, price date, and conversion	1	1	1	1	1	1	1	1	1
Choice of model	0	1	1	1	0	1	1	1	1
Assumptions	1	1	1	1	1	1	1	1	1
Analytical methods	1	1	1	1	1	1	1	1	1
Study parameters	1	1	1	1	1	1	1	1	1
Incremental costs and outcomes	1	1	1	1	1	1	1	1	1
Characterizing uncertainty	1	1	1	1	1	1	1	1	1
Characterizing heterogeneity	0	0	0	0	0	0	1	1	0
Study findings, limitations, generalizability, and current knowledge	1	1	1	1	1	1	1	1	1
Source of funding	1	1	1	1	1	1	1	1	1
Conflicts of interest	1	1	1	1	1	1	1	1	1
Total	21	23	23	21	22	20	21	23	21
Ratio	0.88	0.96	0.96	0.88	0.92	0.83	0.88	0.96	0.88
Quality	Good	Good	Good	Good	Good	Good	Good	Good	Good
**Section item**	**Hoyo et al**. **(****30****)**	**Modi et al**. **(****39****)**	**Lowry et al**. **(****14****)**	**Witt Udsen et al**. **(****34****)**	**Romero-Sanchiz et al**. **(****35****)**	**Lugo et al**. **(****9****)**	**Oksman et al**. **(****20****)**	**Fusco and Francesco** **(****31****)**	**Lopez-Villegas et al**. **(****37****)**
Title	1	1	1	1	1	1	1	1	1
Abstract	1	0	1	0	1	0	0	1	1
Background and objectives	1	1	1	1	1	1	1	1	1
Target population and subgroups	1	0	0	1	1	0	0	0	0
Setting and location	1	1	1	1	1	1	1	0	1
Study perspective	1	1	0	1	1	1	0	1	1
Comparators	1	1	1	1	1	1	1	1	1
Time horizon	1	1	1	1	1	1	1	1	1
Discount rate	1	1	1	1	1	0	0	1	1
Choice of health outcomes	1	1	1	1	1	1	1	1	1
Measurement of effectiveness	1	1	1	1	1	0	0	1	0
Measurement and valuation of preference based outcomes	1	1	1	1	1	1	1	1	1
Estimating resources and costs	1	1	1	1	1	1	0	1	1
Currency, price date, and conversion	1	1	1	1	1	0	0	1	1
Choice of model	0	1	1	1	1	1	0	1	0
Assumptions	1	1	1	1	1	1	0	1	1
Analytical methods	1	1	1	1	1	1	1	1	1
Study parameters	1	1	1	1	1	1	1	1	1
Incremental costs and outcomes	1	1	1	1	1	1	1	1	1
Characterizing uncertainty	1	1	0	1	1	0	1	1	1
Characterizing heterogeneity	0	0	0	1	1	1	1	1	1
Study findings, limitations, generalizability, and current knowledge	1	1	1	1	1	0	1	1	1
Source of funding	1	1	1	0	1	1	1	1	1
Conflicts of interest	1	1	0	1	1	1	1	1	1
Total	22	21	19	22	24	18	15	22	21
Ratio	0.92	0.88	0.79	0.92	1	0.71	0.63	0.92	0.88
Quality	Good	Good	Fair	Good	Excellent	Fair	Average	Good	Good
**Section item**	**Wan et al**. **(****10****)**	**Watson et al**. **(****32****)**	**Buvik et al**. **(****41****)**	**Oostingh et al**. (12)	**Yoo et al**. **(****33****)**	**Krishnan et al**. **(****8****)**	**Bahrainwala et al**. **(****15****)**	**de Jong et al**. **(****29****)**
Title	1	1	1	1	1	1	1	1
Abstract	0	0	0	1	1	0	0	1
Background and objectives	1	1	1	1	1	1	1	1
Target population and subgroups	0	0	0	0	0	1	0	1
Setting and location	1	0	1	0	1	0	1	1
Study perspective	0	1	1	1	1	1	1	1
Comparators	1	1	1	1	1	1	1	1
Time horizon	1	1	1	1	1	1	1	1
Discount rate	0	0	1	0	1	1	1	0
Choice of health outcomes	1	1	1	1	1	1	1	1
Measurement of effectiveness	1	1	1	1	1	1	1	1
Measurement and valuation of preference based outcomes	1	1	1	1	1	1	1	1
Estimating resources and costs	1	0	1	0	1	1	1	1
Currency, price date, and conversion	1	1	0	1	1	1	1	0
Choice of model	1	1	0	1	1	1	1	1
Assumptions	1	1	1	1	1	1	1	1
Analytical methods	1	1	1	1	1	1	1	1
Study parameters	1	1	1	1	1	1	1	1
Incremental costs and outcomes	1	1	0	1	1	1	1	1
Characterizing uncertainty	0	0	1	1	1	1	1	1
Characterizing heterogeneity	1	0	0	0	0	0	0	0
Study findings, limitations, generalizability, and current knowledge	1	1	1	1	1	1	1	1
Source of funding	1	1	1	1	0	1	1	1
Conflicts of interest	1	1	1	1	1	1	1	1
Total	19	17	18	19	21	21	21	21
Ratio	0.79	0.71	0.75	0.79	0.88	0.88	0.88	0.88
Quality	Fair	Fair	Fair	Fair	Good	Good	Good	Good

The studies were graded on the bases of the number of items accomplished and classified as follows:

- Excellent, if all items were present in the study: only one study (3%) reported (35).
- Good, if at least 80% of the items were satisfied: 24 studies (69%) reported (8, 11, 12, 15, 17–19, 21–31, 33, 34, 37–40).
- Fair, if at least 70% of the items were satisfied: nine studies (26%) reported (7, 9, 10, 12, 14, 16, 32, 36, 41).
- Average, if at least 60% of the items were satisfied: one study (3%) reported (20).

The five items most likely to not be reported were: Characterizing heterogeneity (11, 31%); Target population and subgroups (13, 37%); Abstract (22, 63%); Choice of model (24, 69%); Discount rate (25, 71%).

In contrast, seven CHEERS checklist items were fulfilled by all studies (100%): Title; Background and objectives; Comparators; Time horizon; Choice of health outcomes; Analytical methods; Study parameters.

All the other 12 CHEERS items were included in almost 80% of the studies: Measurement of effectiveness (28, 80%); Currency, price date, and conversion (28, 80%); Setting and location (29, 83%); Characterizing uncertainty (30, 86%); Study perspective (32, 91%); Measurement and valuation of preference-based outcomes (32, 91%); Estimating resources and costs (32, 91%); Source of funding (32, 91%); Assumptions (33, 94%); Incremental costs and outcomes (34, 97%); Study findings, limitations, generalizability, and current knowledge (34, 97%); Conflicts of interest (34, 97%).

## Type of technologies or interventions for digital health innovation

A summary of findings and evidence found is reported in Table 4.

**Table 4:** Summary of findings.

**Author (published year)**	**Intervention**	**Type of intervention**	**Cost measurement (incremental cost)**	**Consequence measurement (incremental effect)**	**ICER**	**Key findings (main conclusion)**
Bahrainwala et al. (2020) (15)	Drone Observed Therapy System (DrOTS) intervention inludes: (i) drones to deliver sputum samples and tuberculosis (TB) medication; (ii) GeneXpert™ MTB/RIF (Cepheid, Sunnyvale CA USA) molecular platform to increase sensitivity and specificity of TB diagnosis; (iii) WHO endorsed evriMED™ (Wisepill, Somerset West, South Africa) digital adherence monitoring technology to remotely assess TB treatment adherence by monitoring daily opening of an electronic pill box.	Digital technologies and innovations	The incremental cost per additional TB patient diagnosed in DrOTS was 2,631$	There was a 61.2% (95% CI 58.1–65.2, *P* < 0.05) increase in case finding and treatment initiation over usual care. With the implementation of digital adherence monitoring technologies, those outcomes were respectively 405 (91.0%) and 40 (9.0%) in DrOTS. This represents a 2.6% (95% CI −1.8 to 7.5, *P* = 0.47) increase in successful outcomes	DrOTS has an ICER value of $177 per DALY averted compared to usual care for diagnosis and treatment of TB	Innovative technology packages including drones, digital adherence monitoring technologies, and molecular diagnostics for TB case finding and retention within the cascade of care can be cost effective. Their integration with other interventions within health systems may further lower costs and support access to universal health coverage
Prinja et al. (2018) (25)	ReMiND (REducing Maternal and Newborn Deaths), a mHealth application that tracks and supports clients for the Accredited Social Health Activist (ASHA) workers and provides inputs for individualized service and counseling needs	Digital technologies and innovations	From societal perspective, there was a cost saving of USD 425 million with ReMiND intervention	The implementation of ReMiND from 2011 to 2020 would save 4,127,529 DALYs	From societal perspective, intervention resulted in a cost saving of USD 90 per DALY averted and USD 2,569 per death averted. From health system perspective, the intervention determined an incremental cost of INR 12,993 (USD 205) per DALY averted and INR 371,577 (USD 5,866) per death averted	Findings of our study suggest strongly that the mHealth intervention as part of the ReMiND intervention is very cost effective from Indian health system's viewpoint, and cost saving from a societal perspective, and should be considered for replication elsewhere in India
Lowry et al. (2020) (14)	Digital Breast Tomosynthesis (DBT), a 3d diagnostic imaging system	Digital technologies and innovations	The transition from conventional Digital Mammography (DM) to DBT increased total costs by $395,553–445,722 per 1,000 screening-eligible women	In the base case analysis, breast cancer mortality and life-years were overall consistent between the DBT and DM screening scenarios. Small QALY gains were seen with DBT compared to DM, with incremental gains ranging from 1.97 to 3.27 per 1,000 women	The ICERs for DBT relative to DM ranged from $195,026–270,135 per QALY gained	DBT reduces false-positive exams while achieving similar or slightly improved health benefits. At current reimbursement rates, the additional costs of DBT screening are likely high relative to the benefits gained; however, DBT could be cost-effective at lower screening costs
Krishnan et al. (2019) (8)	Shape intervention offered: (a) tailored behavior change goals; (b) skills training materials; (c) weekly interactive voice response telephone calls; (d) monthly telephone coaching from a registered dietitian; (e) a no-cost 12-month membership to a facility of their choice	Telephone support	The incremental cost of Shape relative to usual care was US $758	The primary measure of effectiveness in the trial was weight change from baseline to 12 months. Weight change was converted into a health-related quality of life change score. Mean difference in weight change of the intervention and usual CARE arms with regard to baseline approached statistical significance at the 12-month (−1.4 kg [−2.8 to −0.1]) assessment. The difference in weight change across arms was transformed to QoL change scores for Shape participants and usual care participants (+0.009 and +0.005, respectively)	In the base case, the ICER was of US $55,264 per QALY gained	Shape intervention is cost-effective based on established benchmarks, indicating that it can be a part of a successful strategy to address the nation's growing obesity epidemic in low-income at-risk communities
Levy et al. (2017) (21)	Phone conversation with a tobacco treatment specialist (TTS) about smoking cessation counseling. In addition, the TTS offered to connect patients with social services *via* HelpSteps.com, a web-based clearing-house for local social services relevant to low-income individuals	Telephone support	The incremental cost per additional quit is $4,137 (95% CI $2,671– $8,460) over the 20-month study period	Comparing intervention to usual care, we estimate a risk difference of 9.7%, or approximately 69 (95% CI 33–108) incremental quits (9.7% × 707 smoker participants) based on the intention to treat analysis	The overall incremental cost per additional life year saved is $7,301 (95% CI $4,545–$15,400)	The proactive population-based smoking cessation program tested in Project CLIQ under conservative assumptions did not appear as cost-effective as a related strategy, but demonstrated favorable cost-effectiveness compared to other smoking cessation programs and is likely to be highly cost-effective by common cost-effectiveness thresholds ($50,000–$150,000/additional quality-adjusted life year) compared to other health interventions
Romero-Sanchiz et al. (2017) (35)	Internet-based Cognitive-Behavioral Therapy intervention program (“Smiling is Fun”) for depression with or without psychotherapist support	Web platforms and digital health portals	The totally self-guided (TSG) Internet-based program led to save USD 644.11 per patient in comparison with improved treatment as usual (iTAU)	The effectiveness was measured as reduction of Beck Depression Inventory II (BDI-II), and totally self-guided Internet-based program led to a reduction of 3.80 point in comparison with improved treatment as usual	The complete case analyses revealed an incremental cost-effectiveness ratio (ICER) of €−169.50 for the TSG group compared with iTAU	The results of this study indicate that Internet-based CBT interventions are appropriate from both economic and clinical perspectives for depressed patients in the Spanish primary care system. These interventions not only help patients to improve clinically but also generate societal savings
Watson et al. (2018) (32)	Internet-based cognitive-behavioral therapy for bulimia nervosa (CBT-BN)	Web platforms and digital health portals	The average cost per abstinent patient at 1-year follow-up was $16,777 (95% CI = $10,298, $27,042) for face-to-face and $14,561 (95% CI = $10,165, $21,028) for Internet-based CBT-BN	The primary outcome of abstinence for Internet-based CBT-BN was inferior to face-to-face CBT-BN at post-treatment but non-inferior at 1-year follow-up QALY gain: over the course of treatment, participants in each group gained on average ~1 week of full health. At the end of one year, those in face-to-face had gained 4 weeks of full health and those in Internet-based gained 5 weeks. The clinical significance of these differences are small	Not mentioned	Cost-effectiveness of Internet-based CBT-BN is comparable with that of an accepted standard. Internet-based dissemination of CBT-BN may be a viable alternative for patients geographically distant from specialist eating disorder services who have an unmet need for treatment
Nordyke et al. (2019) (27)	Implementation and use of software to treat disease in Type 2 Diabetes and Hypertension patients	Web platforms and digital health portals	Average Health resource utilization (HRU) savings ranged from $97 to $145 per patient per month	Not reported	At a willingness-to-pay threshold of $50,000/QALY, the intervention is estimated to be cost effective at total 3-year program costs of $6,468 (T2DM)	The Digital therapeutics studied may provide substantial cost savings, in part by reducing the use of conventional medications. Clinical inertia may limit the full cost savings of digital therapeutics
Sharifi et al. (2017) (40)	Study of Technology to Accelerate Research (STAR) intervention is a electronic health records (EHRs) modified to facilitate childhood obesity management by prompting diagnosis and providing decision support and electronic resources for evaluation, management, and follow-up care	Web platforms and digital health portals	Over 10 years, the intervention would cost $239 million or $119 per child reached	Relative to usual care, the intervention could reduce mean per capita BMI by 0.5 U among those reached	It is estimated an intervention cost of $237 per BMI unit reduced. At 10 years the intervention would avert 42,900 cases of obesity and 226,000 lifeyears with obesity at a net cost of $4,085 per case and $774 per year with obesity averted	This childhood obesity intervention with electronic decision support for clinicians and self-guided behavior-change support for parents may be more cost-effective than previous clinical interventions
Oostingh et al. (2019) (12)	Smarter Pregnancy, a mHealth coaching program in addition to the usual care in women of subfertile couples who start their first *in vitro* fertilization (IVF) cycle	Text messaging intervention	From health care perspective, intervention led to save €206.300, in comparison to usual care From societal perspective, intervention led to save €270.000, in comparison to usual care	Measure of effectiveness was expressed as the number of ongoing pregnancies after two IVF cycles and the use of the mHealth program resulted in 86 additional pregnancies	The ICERs from health care and societal perspectives per additional ongoing pregnancy equaled –€2,250 (95%CI −3,030 to −760) and –€3,050 (95% CI −3,960 to −540), respectively	The mHealth coaching program Smarter Pregnancy is potentially cost saving for subfertile couples preceding their first IVF treatment with low costs and promising cost-effectiveness estimates
Jo et al. (2019) (23)	mCARE package, a mobile phone-based system to improve communication and coordination between community health providers and the pregnant women they serve	Text messaging intervention	Overall, the total incremental cost of the comprehensive mCARE group compared to the basic mCARE group is estimated as $319,000 over the two years of implementation	Once adjusting for a population of 1 million, it was estimated a difference of 354 (uncertainty range 145–571) newborn deaths averted between the intervention and comparison groups	The comprehensive mCARE group (with SMS and home visit reminders) was highly cost-effective compared to the basic mCARE group with $901 per death averted and $31 per DALY averted	Study findings suggest that the addition of SMS and home visit reminders based on a mobile phone-facilitated pregnancy surveillance system may be cost-effective. Incorporating mHealth strategies such as SMS and home visit reminders to proven community-based delivery strategies may improve service utilization and program cost effectiveness in lowresource settings
Cleghorn et al. (2019) (24)	Intervention was a national mass media promotion of selected smartphone apps for weight loss compared with no dedicated promotion	Text messaging intervention	Costs to the health system of New Zealand was $2.3 million over the lifetime of the modeled population	The estimated impact of the base-case intervention was a health gain of 29 QALYs	Costs per QALY gained (or the incremental cost-effectiveness ratio) were NZ $79,700 (US $53,600) for the standard download rate	The mass media promotion of a smartphone app for weight loss produced relatively small health gains on a population level and was of borderline cost-effectiveness for the total population
O'Sullivan et al. (2020) (26)	Intervention was a “healthy lifestyle package,” including dietary and exercise advice and a smartphone app to reinforce health messages to reduce the incidence of gestational diabetes mellitus	Text messaging intervention	There were no significant differences across intervention and control groups in mean cost of antenatal admissions, delivery costs or total health care utilization	Women in the intervention group lost fewer QALYs, though the difference was not statistically significant (2.75 vs. 2.85, *P* = 0.38)	The ICER for QALYs was €2,914 per QALY gained	Providing a mobile health-supported lifestyle intervention to pregnant women with an elevated BMI may be a cost-effective way of improving maternal and infant health
Islam et al. (2020) (11)	Text messaging intervention plus standard-care for patients with type 2 diabetes	Text messaging intervention	The calculation of the incremental costs showed that the text messaging intervention can be delivered at costs of 24 Int.$ per patient	A statistically significant difference in HbA1c was observed in favor of the intervention group	ICER of 38 Int.$ per 1% reduction in HbA1c and of 2,406 Intl.$ per QALY gained	The mobile phone text-messaging is an effective and cost-effective method in improving glycemic control Text-messaging might be a valuable addition to standard treatment for diabetes care in low-resource settings
Sjostrom et al. (2017) (38)	Mobile app Tät®, a treatment program focused on pelvic floor muscle training (PFMT), and information about stress urinary incontinence and lifestyle factors	Mobile phone-based systems and applications	The total cost per participant was higher in the app group (€547.0) than that in the control group (€482.4)	In the app group, there was significant improvement in QoL at follow-up. In contrast, the control group did not display a significant reduction in scores. In app group, 0.01006 QALY gain. In control group, 0.00158 QALY gain	The incremental cost effectiveness ratio was of €7,615.5 per QALY in the base case scenario	The app for treating stress urinary incontinence is a new, cost-effective, first-line treatment with potential for increasing access to care in a sustainable way for this patient group
Kumar et al. (2018) (22)	Mobile cognitive behavioral therapy (CBT) program for Generalized anxiety disorder (GAD) with e-learnings and techniques to help them manage their anxiety and receive individualized support from a coach over a 3-month program	Mobile phone-based systems and applications	From a payer perspective, mobile CBT reduces overall costs by approximately $339 million when compared to traditional CBT	Mobile CBT led to a gain of 34,108 QALYs when compared to traditional CBT and 81,492 QALYs when compared to the status quo	Incremental cost effectiveness of intervention when compared to traditional cognitive behavioral therapy was of 65,380 $/QALY Incremental cost effectiveness when compared to status quo was of 54,606 $/QALY	Mobile CBT may lead to improved health outcomes at lower costs than traditional CBT or no intervention and may be effective as either prevention or treatment
Modi et al. (2020) (39)	Innovative Mobile Technology for Community Health Operation (ImTeCHO), a job aid for staff of primary health centers to increase the coverage of maternal, neonatal, and child health MNCH care	Mobile phone-based systems and applications	The implementation of ImTeCHO resulted in an annual incremental cost of US $163,841	Implementation of the ImTeCHO intervention resulted in 11 infant deaths per 1,000 live births averted in the per-protocol analysis. This implies a reduction of 16% infant deaths per-protocol in the study area. This resulted in an increase in 735 life years, with a life expectancy of 68.35 years	ImTeCHO is a cost-effective intervention from a program perspective at an incremental cost of US $74 per life-years saved or US $5,057 per death averted	The findings of the study strongly suggest that the mHealth intervention as part of the ImTeCHO program is cost-effective and should be considered for replication elsewhere in India
Song et al. (2018) (13)	Smartphone application named “Karada-no-kimochi“. The user can record their menstrual dates, basal body temperatures, and their mental and physical disorders. The application predicts the menstrual cycle, i.e. it predicts the next day of bleeding, the length of the menstruation period, and the ovulation day	Mobile phone-based systems and applications	The total cost of expenses, loss of productivity and application fee was less for the intervention group than for the control group by JPY 134,000 (USD 1,170) in total	The QALY in the intervention group was 6.84, which is 0.07 higher than that in the control group (6.77)	Incremental cost effectiveness of intervention when compared to traditional was of 1,914,285 JPY (USD 16,714) per QALY	This RCT study suggested that the use of “Karadano- kimochi” may be effective in reducing the onset of dysmenorrhea and depression. The cost-effectiveness analysis indicated a dominant result from the use of the application
Whetten et al. (2018) (17)	Telehealth platform that includes rapid radiograph image transfer and two-way audiovisual capacity, as well as report generating capacity. This enables consulting neurosurgeons and neurointensive care specialists to review imaging and talk with/examine the patient and generate a report	Video-conferencing system	The use of ACCESS led to save $4,241 ($3,952–$4,438) per patient	Intervention, in comparison with usual care, increased QALYs by 0.20 (0.14–0.22)	Incremental cost effectiveness when compared to traditional care was of $-21,205 per QALYs	The teleneurology program ACCESS is a cost-effective approach to managing patients with neuro-emergent conditions in rural areas. In addition to providing financial benefits, a teleneurology program produces better patient outcomes, and offers societal benefits through reduction of stroke related disability and increased convenience to patient's families
Yoo et al. (2016) (33)	Introduction of telemedicine in the Intensive Care Unit (ICU)	Video-conferencing system	Incremental cost of $516 per patient compared with ICU without telemedicine	The incremental effect in the intervention group was of 0.011 (0.005–0.017) QALYs	Incremental cost-effectiveness ratio was of $45,320 per QALY	Telemedicine in the ICU is cost-effective in most cases and cost saving in some cases
Thakar et al. (2018) (16)	Telemedicine consultation center	Video-conferencing system	The mean per episode cost was INR 2,338 (38.0 USD) for TeleMedicine (TM) care vs. INR 5,479 (89.o USD) for routine care. Intervention resulted to be cost saving	The effectiveness of telemedicine care was calculated using efficiency in terms of the percentage of successful TM consultations. The overall effectiveness of the TM-care group was 917.4 and that of routine care was 132.8	The ICER value was calculated to be −34,900 INR (571.9 USD)/unit of effectiveness (2,338 −5,479 [38.3–89.8]/0.89–0.80)	TM care dominates the in-person care strategy by providing more effective and less expensive follow-up care for a remote post–neurosurgical care population in India
Buvik et al. (2019) (41)	Telemedicine consultations using real-time videoconferencing	Video-conferencing system	In comparison to routine care, the intervention produced an annual cost savings of €19,500 (USD 16,516)	The average QALYs gained per patient in the telemedicine group was .09 which was not significantly different to the .05 gain in the standard consultation group, *P* = 0.29	Not mentioned	Video-assisted orthopedic consultations, rather than having patients travel to the specialist hospital for consultations, is cost-effective from both a societal and health sector perspective
**Fusco and Francesco (2016)** **(****31****)**	Standard Rehabilitation + Telerehabilitation after total knee replacement	Video-conferencing system	Intervention on average led to save $263 (95% CI –$382 to –$143) per person	The incremental effect was measured by the knee flexion range of motion (ROM) gained and by QALY gained	The ICER (adopting Ita-NHS perspective) is –€960 ($1,352)/QALY [ceiling ratio: €30,000 ($42,200)/QALY]	The analysis suggested the intervention to be cost-effective, even less expensive and more effective
Vestergaard et al. (2020) (19)	Telehealthcare solution (TeleCare North Heart Failure) in heart failure patients as add-on to usual care	Video-conferencing system	Telemedicine reduced total healthcare costs by 35% [5,668 ($7,557) off a base of 16,241 British Pounds Sterling ($21,654)]	The 1-year adjusted QALY difference between the telehealthcare solution and the usual care group was 0.0034 (95% CI: −0.0711 to 0.0780), indicating an insignificant gain in health-related quality of life (HRQoL) for patients receiving the tele-healthcare solutio	Based on the incremental cost and QALY estimates and an assumed cost-effectiveness threshold of £20,000 ($ 26,666) per QALY,27 the telehealthcare solution provides a positive incremental net monetary benefit (NMB) of £5,164 ($ 6,885)/QALYs	All scenario analyses showed the same result with telehealthcare associated with lower costs and an insignificant impact on patients' HRQoL
Painter et al. (2017) (28)	Telemedicine Outreach for Post-Traumatic Stress Disease intervention involving offsite PTSD care teams located at parent VAMCs to support on-site CBOC providers	Video-conferencing system	The overall incremental cost of the intervention was $2,495 (p < .01) per patient	The total QALY gain from intervention is 0.008 compared to usual care	The primary analysis resulted in a median ICER of $185,565 per QALY (interquartile range $57,675 to $395,743)	Because of the upfront training costs and the resource-intensive nature of the intervention, associated expenses were high. Although PTSD-specific effectiveness measures were significantly improved, these changes did not translate to QALYs in the main analysis
Wang et al. (2016) (36)	Telemedicine Center at the West China Hospital (TCWCH) program intervention (a digital network with video equipment and image transfer that can be used in simultaneously conducting longdistance education or consultation)	Video-conferencing system	Telemedicine network resulted in an estimated net saving of $2,364,525 (if the patients traveled to the hub) or $3,759,014 (if the specialists traveled to the spoke hospitals)	It is a cost-saving analysis, there is no clinical measurement	There is no ICER	The intervention was highly cost saving
Clarke et al. (2018) (7)	National Health Service Direct Telehealth program (that included the planning and administration of the program, developing operating policy and procedures and technical requirements, developing clinical process workflow for the call center, and reporting and management of data elements for evaluation)	Video-conferencing system	The average saving was £1,023 ($1,280) per patient per year	Measure of effectiveness was the resource utilization data obtained from multiple sources, including A&E visits, ambulance usage, and hospitalization	Data did not include quality of life, and so we were unable to undertake cost/benefit analysis	The wide variance on savings and the uncertainty of monitoring cost do not allow a definitive conclusion on the cost-effectiveness as an outcome of this study
Witt Udsen et al. (2017) (34)	Telehealthcare solution and monitoring by a community-based healthcare team, in addition to usual care for patients with chronic obstructive pulmonary disease	Video-conferencing system	The base-case adjusted mean difference in total costs between telehealthcare and usual care was €728 (967 USD) [95% CI −754 to 2,211 (1,001–2,936)]	The adjusted mean difference in quality-adjusted life-years gained was 0.0132 (95% CI −0.0083 to 0.0346)	The ICER is €55.327 (73,769 USD) per QALY	Telehealthcare is unlikely to be a cost- effective addition to usual care, if it is offered to all patients with chronic obstructive pulmonary disease and if the willingness-to-pay threshold values from the National Institute for Health and Care Excellence are applied
Lugo, et al. (2019)	An out-of-hospital Virtual Sleep Unit (VSU) based on telemedicine to manage all patients with suspected OSA	Video-conferencing system	Intervention on average led to save 153.34 € (181.04 USD)	The incremental effectiveness was estimated in 0.0108 QALYs	Not mentioned	The VSU offered a cost-effective means of improving QALYs than routine care. Our findings indicate that VSU could help with the management of many patients, irrespective of CPAP use
Nguyen et al. (2016) (18)	A Telemedicine Program, called Singapore Integrated Diabetic Retinopathy Program (SiDRP), that provides “real-time” assessment of diabetic retinopathy photographs by a centralized team of trained and accredited graders supported by a tele-ophthalmology information technology infrastructure	Video-conferencing system	Intervention (SiDRP) generates a cost savings of $173 per patient	The total QALY gain from the SiDRP is almost the same as the routine care model (i.e., 13.1129 vs. 13.1123 QALYs)	$-288.333 per QALYs	The SiDRP model saves costs compared with the traditional model. This provides evidence in support of extending the SiDRP model across Singapore and outside the public sector
de Jong et al. (2020) (29)	Telemedicine with myIBDcoach (my Inflammatory Bowel Disease coach)	Video-conferencing system	The intervention resulted in a mean annual cost saving of €547 (612 USD) per patient [95%CI €-1,029 to €2,143 (1,152–2,400 USD)]	Patients in the intervention group showed a mean gain in quality adjusted life years (QALY) of 0.002 (95%CI, [-0.022, 0.018])	Not explicited	Telemedicine with myIBDcoach is cost saving and has a high probability of being cost effective for patients with IBD
Wan et al. (2019) (10)	A combination of telemedicine and shared medical appointments in transition-age young adults with Type 1 Diabetes	Video-conferencing system	There was no significant difference in total costs	No significant differences in 9-month quality-adjusted life; however, the control group had a larger decline from baseline in utility than the intervention group, indicating a quality of life (QoL) benefit of the intervention (difference in difference mean ± SD: 0.04 ± 0.09; *P* = 0.03)	No within-trial incremental cost-effectiveness ratio was calculated due to the lack of significant difference in 9-month total costs or QALYs	The intervention (CoYoT1) care model may help young adults with T1D maintain a higher QoL with no increase in costs
Oksman et al. (2017) (20)	A tele-based health-coaching intervention among patients with type 2 diabetes (T2D), coronary artery disease (CAD) and congestive heart failure (CHF)	Video-conferencing system	The incremental cost for intervention in comparison with control was of 432€ (488USD) [−135€ to 999€ (−153USD to 1,128USD)]	The cost-effectiveness plane for HRQoL (15D) after health coaching showed that the intervention was more effective compared to care as usual [0.009 (0.000–0.018)]	The overall incremental ICER was €48,000 (54,237 USD) per QALY	Based on the results of this study, health coaching improved the QoL of type 2 diabetes and coronary artery disease patients with moderate costs. However, the results are grounded on a short follow-up period, and more evidence is needed to evaluate the long-term outcomes of health-coaching programs
Lopez-Villegas et al. (2020) (37)	Telemonitoring (TM) of patients with pacemakers in comparison with conventional monitoring (CM)	Video-conferencing system	Incremental costs per patient included in the TM vs. CM group constituted €1,807.87 (USD 2,006.52) [CI: −646.99 to 4,262.73 (−718.08 to 4,731.11)] from the perspective of the NHS and €1,865.52 (USD 2,070.50) [CI: −608 to 4,335.25 (674.81–4,811.6)] including patient/family cost	This study provided evidence showing that 12 months after pacemaker implantation, health-related quality of life was similar between groups of RM and conventional follow-up in hospital	The mean ICER amounted to €53,345.27 (USD 59.206,38) from the perspective of the NHS or €55,046.40 (USD 61.094.78) including patient/caregiver costs	Cost–utility analysis of TM vs. CM shows inconclusive results because of broad confidence intervals with ICER from potential savings to high costs for an additional QALY, with the majority of ICERs being above the usual NHS thresholds for coverage decisions
Hoyo et al. (2019) (30)	Telemonitoring of Crohn's Disease and Ulcerative Colitis (TECCU) Web platform for telemonitoring complex inflammatory bowel disease and nurse-assisted telephone care	Video-conferencing system	TECCU determined a median cost reduction from a societal perspective of €211 (US $231) per patient (95% CI €−600 to 180 per patient; US $-657 to 197 per patient)	The incremental efficacy of TECCU was 0.19 (0.33–0.14) relative to control (median incremental efficacy calculated with the bootstrapping procedure was 0.21, 95% CI −0.07 to 0.66)	TECCU vs. control estimated a median ICER of €−1,005 (95% CI €−13,518 to 3,137; US $1,100, 95% CI US $-14,798 to 3,434)	There is a high probability that the TECCU Web platform is more cost-effective than standard and telephone care in the short term

### Video-conferencing system

Video conferencing systems consist in programs that allow the delivery of specialist consultations *via* video for remote patients with any kind of condition or disease.

In this review, a total of 17 of the included studies evaluated the cost-effectiveness of video conferencing systems (7, 9, 10, 16–20, 28–31, 33, 34, 36, 37, 41).

The disciplines examined were the following: (1) two studies focused on a video conferencing system applied in a teletrauma/telestroke context, delivering neurology care to remote patients (16, 17); a network of audiovisual communications and data systems allowed to link hospital intensive care units to intensivists and other critical care professionals at remote locations (33); (2) two studies compared a standard rehabilitation program to a telerehabilitation program for patients that aimed to improve access and quality of care, avoid patient travel, and reduce health care costs (31, 41); (3) two videoconferencing services for general practices consisted of telehealthcare equipment for continuous monitoring of physiological measurements (19, 36); telemedicine outreach for Post-Traumatic Stress Disease (PTSD) examined the impact of telemedicine-based collaborative care for PTSD with enhanced usual care without on-site psychiatrists (28); (4) three studies focused on the effects of a videoconferencing system for patients with chronic obstructive pulmonary disease (7, 9, 34); (5) three studies implemented telemedicine interventions for patients with diabetes (10, 18, 20); (6) two video conferencing platforms were used for telemonitoring complex inflammatory bowel disease compared to standard care (29, 30); (7) one study examined patients with implanted pacemakers who received home monitoring with internet-based remote monitoring service and video-consultation service (37).

Two out of the 17 articles included in this section reported not to be cost-effective (28, 37).

The remaining 15 articles stated the digital health interventions to be cost-effective: video conferencing systems gained higher QALYs with cost-saving in 9 (7, 9, 16–18, 29–31, 41); video conferencing systems gained QALYs with higher cost at an acceptable ICER in 6 (10, 19, 20, 33, 34, 36).

### Text messaging intervention

Text message-based health interventions provide patients with reminders, education or self-management assistance for a broad spectrum of health conditions.

In this review, the text messaging interventions concerned the following topics: (1) a mobile phone text messaging program for people with type 2 diabetes mellitus was implemented in Bangladesh (11); (2) Smarter Pregnancy, a text messaging coaching program in addition to the usual care for women of subfertile couples who start their first *in vitro* fertilization cycle (12); (3) mCARE package, a short message service and home visit reminders sent to pregnant women to promote the care-seeking of essential maternal and newborn care services (23); (4) a New Zealand national mass media promotion of selected smartphone apps with text messaging service for weight loss (24); (5) the Pregnancy, Exercise And nutrition Research study (PEARs) intervention, a ‘healthy lifestyle package,' that included dietary and exercise advice and text messages to reinforce health reminders (26).

All five of the included studies in this category found that text messaging interventions were cost-effective. Intervention groups gained higher QALYs with cost-savings in one study (12); intervention groups gained QALYs with slightly higher cost at an acceptable ICER in the other four studies (11, 23, 24, 26).

### Web platforms and digital health portals

A digital health portal is a secure online web portal that gives patients convenient, 24-h access to personal health information from anywhere *via* an Internet connection, often “tethered” to their integrated electronic health records.

In this review, the web portals for citizens and patients retrieved during the screening process are the following: (1) “Smiling is fun”, an Internet-delivered, self-help web portal for the treatment of depression, consisting of 10 cognitive behavioral therapy modules to cope with depression (35); (2) an e-Health portal that gives internet-based cognitive-behavioral therapy designed for patients with bulimia nervosa (32); (3) a web portal that provides digital behavioral and lifestyle intervention for type 2 diabetes mellitus and hypertension patients (27); (4) an electronic health record (EHR)-based decision support program for parents with 6- to 12-year-old children with obesity, providing behavioral therapy and support (40); and (5) Tät service, a web portal support service designed to inform patients on stress urinary incontinence, that provides a pelvic floor muscle training program and prescribes pelvic floor muscle training 3 times daily during treatment (38).

The cost-effectiveness acceptability analysis indicated improved health outcomes with similar, or even lower costs for all the 5 studies of this section (27, 32, 35, 38, 40).

### Telephone support

Telephone support is the use of phone calls by specialists, such as nurses, doctors and healthcare professionals in general, to deliver self-care support and/or management.

Studies selected in this category focused on the following topics: (1) a tobacco treatment telephone support program, that provides smoking cessation counseling to participants in the intervention group (21); and (2) Shape Program, an adaptive telephone-based coaching system, designed to prevent weight gain in black female primary care patients that consists of personalized obesogenic behavior change goals assigned every 2 months, a tailored skills training curriculum, patient self-monitoring delivered *via* a fully automated interactive voice response system and 12 counseling calls with a registered dietitian (8).

All of the studies in this section reported greater gains in quality-adjusted life years at a similar or slightly higher cost, resulting in cost-effectiveness based on established benchmarks (8, 21).

### Mobile phone-based systems and applications

Mobile phone-based applications include all of the services and systems that provide support, delivery and promotion of care through the monitoring and sharing of health information *via* mobile technology, such as wearables and health tracking apps.

Mobile phone-based applications retrieved in this review included: (1) a mobile cognitive-based therapy program set to provide learnings and techniques to help users manage their anxiety (22); (2) Karada-no-kimochi, a mobile application that predicts the menstrual cycle based on recorded data and provides information regarding menstruation (13); and (3) Innovative Mobile Technology for Community Health Operation (ImTeCHO), a mHealth-based intervention that enhances health promotion using multimedia and short message reminders to increase coverage of maternal, neonatal, and child health care (39).

All 3 studies declared improved QALYs with lower cost, suggesting the interventions were highly cost effective (13, 22, 39).

### Digital technologies and innovations

This section includes all articles that focused on the cost-effectiveness of digital health interventions that do not fall within any of the above categories, such as experimental digital diagnostic imaging or experimental technologies.

Digital health innovations in this group were represented by: (1) Digital Breast Tomosynthesis (DBT), a new breast imaging modality that reconstructs cross-sectional slices of the breast, minimizing soft-tissue overlap (14); (2) ReMiND, a program that assists healthcare professionals in the early identification, treatment, and rapid referral for appropriate care of any danger signs among pregnant women or neonates (25); and (3) Drone Observed Therapy System (DrOTS), a project to support community-based tuberculosis case finding using drones to deliver sputum samples and tuberculosis medication between rural communities, diagnostics and treatment facilities (15).

Two out of the three studies in this section found the digital health interventions to be highly cost-effective, resulting in higher QALYs with cost-savings (15, 25), while one study reported to be cost-effective but with higher costs (14).

## Discussion

Standardized cost-effectiveness analysis should be used to compare different interventions in terms of their consequences and costs so it can be used as a crucial tool to help decision makers or funders understand if digital health interventions and innovations actually determine an increase of QALYs or DALYs with contained costs (42).

In our systematic review, we opted to exclude studies that utilized digital technologies only to record information without any active participation from healthcare personnel as we wanted to focus on interventions that facilitate the communication between citizen/patient and healthcare staff. However, the number of economic evaluations included in the review is in line with the previous review on cost-utility and cost-effectiveness of telehealth interventions published in 2015, where 35 studies assessed effectiveness, utility and costs (6).

Studies show a broad range of digital interventions, reference population, focus disease and discipline interested. The majority included a comparison of a digital health innovation costs and effectiveness in terms of health-related outcomes vs. standard care. However, some did not report all recommended economic and consequence outcome items. For example, Watson et al. (32), Buvik et al. (41), Wang et al. (36), Lugo et al. (9), and De Jong et al. (29), did not calculate the incremental cost effectiveness ratio, while Nordyke et al. (27), did not indicate the incremental effect of the use of intervention to treat disease in type 2 diabetes and hypertension patients.

The transferability of findings across economic evaluations is based on a rigorous data collection strategy, a coherent methodology, and an explicit description of target population, study design and sample size (43). In our review, over half of the studies were randomized controlled trials which are considered to be of high quality and have a low risk of bias in comparison with other study designs, such as expert opinion studies. Sample size varied significantly and ranged from 50 participants in a study where telemedicine was used to monitor patients with pacemakers compared to conventional monitoring (37) to 4.4 million participants where a text messaging intervention promoted weight loss in all of New Zealand's population (24). One article missed to indicate the sample size (14).

Economic evaluations conducted from a societal perspective are generally preferred since one of the principal objectives of public health is to improve the health and the quality of life of the general population (44). Moreover, economic evaluations based on a fixed budget may lead to suboptimal decisions; they are inconsistent with decisions based on willingness to pay for QALYs and are considered to be of low quality in comparison to societal perspective. Of the 35 studies of the review, 19 articles conducted their economic evaluations from a societal perspective, while only one study did not report the perspective (20). On the contrary, articles with a societal perspective took into account all the losses and expenses, direct and indirect, supported by society as a whole, irrespective of who the benefactors were (e.g. production losses, travel costs, absenteeism, presenteeism, premature death, etc.). Articles with a third party payer perspective encompassed intervention costs, outpatient (incl. general practitioners and specialists) and inpatient services, medication and societal service costs.

Modeling techniques are generally used to predict the effect and the potential cost (or savings) of a determined technology where it is not feasible to wait for lifetime data to validate the cost-effectiveness (42). In our review, several studies used modeling techniques to predict the intervention effect and cost over a long period, or even a lifetime (14, 15, 17, 18, 21, 22, 24, 25, 31, 36, 40, 41). In these cases, it became fundamental to clearly express the uncertainty of the analysis described. All studies selected in the review that used predictive models indicated uncertainty (mostly Monte Carlo simulation, but also one-way and multiway sensitivity analyses, threshold analyses and probabilistic sensitivity analyses).

According to previous reviews (6), “Videoconferencing system” was the most represented digital health intervention type but it was also the one that had the most discordant results: five studies report that the applied technology is not cost-effective or that in any case there is not enough evidence to define the cost-effectiveness (7, 19, 28, 34, 37). Vestegard et al. (19) found that telehealth care was associated with lower costs but had an insignificant impact on patients' HRQoL. Painter et al. (28) underlined effectiveness of the digital measures, but admitted that they did not improve QALYs in the main analysis. Clarke et al. (7) could not give a definitive conclusion on the cost-effectiveness as an outcome of their study due to a wide variance on savings and the uncertainty of monitoring cost. Also Lopez-Villegas et al. (37) had inconclusive results due to broad confidence intervals with ICER from potential savings to high costs for an additional QALY, with the majority of ICERs being above the usual NHS thresholds for coverage decisions.

Witt Udsen et al. (34) through their study revealed that telehealthcare is unlikely to be cost-effective in addition to usual care.

A total of three out of the 35 studies (3/35, 8.6%) were found to not be cost-effective: two studies in the videoconferencing system category (28, 37) and one study in the digital technologies and innovations (14). Of the remaining articles, 12 studies (12/35, 34.3%) found digital health interventions gained QALYs with a higher cost at an acceptable ICER when compared with a relative national benchmark (six studies in videoconferencing systems, four studies in text messaging, two studies in telephone support) (8, 10, 11, 19–21, 23, 24, 26, 33, 34, 36). Finally, a total of 20 out of the 35 studies (20/35, 57.1%) found the digital health interventions gained higher QALYs with cost-savings (nine studies in videoconferencing systems, one study in text messaging, five studies in web platforms and digital health portals, three studies in mobile phone-based systems and applications, two studies in digital health technologies and innovations) (7, 9, 12, 13, 15–18, 22, 25, 27, 29–32, 35, 38–41).

Various benefits of digital tools to rural realities were underlined in different studies (15, 17, 23, 25, 28, 32, 39). This is a very important aspect as it could improve and revolutionize the access to care and quality of treatment for a very large number of patients. Video-conferencing systems offered the greatest advantages in reaching rural areas (17, 23, 28, 36), followed by digital technologies and innovations (15, 25), web platforms and digital health portals (32), mobile phone-based systems and applications (39). The most significant achievements in reaching rural areas were found in the USA (17, 28, 32), followed by India (25, 39), China (36) and Madagascar (15).

It is known that cost-effectiveness is a subjective concept since it depends on the willingness to pay (WTP) for specific outcomes. The decision makers' WTP threshold to establish the cost-effectiveness of intervention differs in the literature. For example, in studies from the United Kingdom, a threshold range of £20,000–£30,000/QALY gained is normally used (45). While in America (46) and Australia (47) they use the amount of 50,000/QALY gained, each in their respective currencies (48).

Some studies analyzed digital health interventions that exceeded the threshold (14, 28, 34, 37) while others had costs that remained below the threshold (7). However, it is crucial to consider that interventions are preferable, even with costs over the threshold, if they improve the outcome with minor or the same costs when compared with standard care.

### Limitations

Firstly, due to the heterogeneity of interventions in the included studies, we could not provide synthetic and general conclusions about costs because most costs were expressed in different values and it was not always possible to make systematic comparisons between them. Secondly, by focusing only on articles written in English, our study may be subject to publication bias and the results should be interpreted appropriately. Thirdly, another source of potential bias - typical of economic studies – is the systematic tendency of including/excluding cost items in the analysis. Therefore, results are driven toward a specific perspective, i.e., the social perspective rather than the health systems' perspective. Finally, even if extensively used to evaluate the quality of economic studies in systematic review of economic evaluations, the CHEERS checklist has structural limitations (e.g. different aspects of the study have the same importance, so the completeness of the abstract and methodological issues such as the choice of the model, the assumptions and the management of uncertainties are given the same relevance).

## Conclusion

Despite a growing interest in investing in digital tools in healthcare, the evidence regarding cost-effectiveness of digital tools in the health sector remains scarce and limited.

Through this review it some evidence was found that digital health interventions can affect cost-effectiveness with a favorable effect both in terms of costs and health outcomes. In particular, the findings showed a positive impact especially for studies that implemented a new mobile application or a web portal intervention. We strongly believe that the findings of this research could be used to better inform and orient health policies. More than half of the studies included report that the use of digital health intervention led to the achievement of a better efficiency and outcomes for patients, the optimization of available human and technological resources and the consistent reduction in the costs of the healthcare services provided. Recognized international examples of digital health practices being successful could be the first step to informing and orienting decision makers to structure a new, evidence-based, digital health maturity.

However, due to the heterogeneity across study methods, cost perspectives, disciplines and diseases involved, the comparison between interventions still remains difficult. Further research based on a standardized approach is needed in order to methodically analyze incremental cost-effectiveness ratios, costs and health benefits.

## Data availability statement

The original contributions presented in the study are included in the article/supplementary material, further inquiries can be directed to the corresponding author.

## Author contributions

AG, FC, GF, and WR: conception or design of the work and final approval of the version to be published. AG: data collection. AG, GF, GT, AM, and VP: articles screening, data analysis, interpretation, and drafting the article. FC, AG, GF, AM, and VP: critical revision of the article. All authors contributed to the article and approved the submitted version.

## Conflict of interest

The authors declare that the research was conducted in the absence of any commercial or financial relationships that could be construed as a potential conflict of interest.

## Publisher's note

All claims expressed in this article are solely those of the authors and do not necessarily represent those of their affiliated organizations, or those of the publisher, the editors and the reviewers. Any product that may be evaluated in this article, or claim that may be made by its manufacturer, is not guaranteed or endorsed by the publisher.

## Appendix 1

### Search string

(“digital health”[Mesh Terms] OR “digital health” OR “telemedicine”[Mesh Terms] OR “telemedicine” OR “e health”[Mesh Terms] OR “e health” OR “electronic health” OR “m health”[Mesh Terms] OR “m health” OR “mobile health” OR “remote consultation” OR “digital transformation” OR “home care services” OR “telenursing” OR “health innovation”[Mesh Terms] OR “telemetry”[Mesh Terms] OR “telehealth”[Mesh Terms] OR “telehealth” OR “telecare” OR “digital care”[Mesh Terms]) AND (“cost analysis”[Mesh Terms] OR “cost analysis” OR “cost benefit”[Mesh Terms] OR “cost benefit” OR “cost efficacy”[All Fields] OR “cost effectiveness”[All Fields] OR “cost consequence”[All Fields] OR “economic evaluation” OR “economic outcome” OR “economic assessment” OR “hta”). Search criteria have been adjusted to each database searched.
